# “Ticking Bomb”: The Impact of Climate Change on the Incidence of Lyme Disease

**DOI:** 10.1155/2018/5719081

**Published:** 2018-10-24

**Authors:** Igor Dumic, Edson Severnini

**Affiliations:** ^1^Mayo Clinic College of Medicine and Science, Rochester, MN, USA; ^2^Division of Hospital Medicine, Mayo Clinic Health System, Eau Claire, WI, USA; ^3^Carnegie Mellon University, Heinz College, 4800 Forbes Ave., Pittsburgh, PA, USA

## Abstract

Lyme disease (LD) is the most common tick-borne disease in North America. It is caused by *Borrelia burgdorferi* and transmitted to humans by blacklegged ticks, *Ixodes scapularis.* The life cycle of the LD vector, *I. scapularis*, usually takes two to three years to complete and goes through three stages, all of which are dependent on environmental factors. Increases in daily average temperatures, a manifestation of climate change, might have contributed to an increase in tick abundance via higher rates of tick survival. Additionally, these environmental changes might have contributed to better host availability, which is necessary for tick feeding and life cycle completion. In fact, it has been shown that both tick activity and survival depend on temperature and humidity. In this study, we have examined the relationship between those climatic variables and the reported incidence of LD in 15 states that contribute to more than 95% of reported cases within the Unites States. Using fixed effects analysis for a panel of 468 U.S. counties from those high-incidence states with annual data available for the period 2000–2016, we have found sizable impacts of temperature on the incidence of LD. Those impacts can be described approximately by an inverted U-shaped relationship, consistent with patterns of tick survival and host-seeking behavior. Assuming a 2°C increase in annual average temperature—in line with mid-century (2036–2065) projections from the latest U.S. National Climate Assessment (NCA4)—we have predicted that the number of LD cases in the United States will increase by over 20 percent in the coming decades. These findings may help improving preparedness and response by clinicians, public health professionals, and policy makers, as well as raising public awareness of the importance of being cautious when engaging in outdoor activities.

## 1. Introduction

Lyme disease (LD) is the most common reportable vector-borne zoonosis in the United States, and its incidence has sharply increased over the last decade. The causative pathogen, spirochete *B. burgdorferi*, is transmitted to humans by a tick vector. The main vector of LD is *I. scapularis* in the northeastern and midwestern Unites States, and *Ixodes pacificus* in the Pacific Northwest [1].

The first evidence of Lyme disease dates back to 1883, when a German physician described acrodermatitis chronica atrophycans, which was later recognized as the late dermatological manifestation of LD [2, 3]. Later on, other seemingly unassociated manifestations were reported such as erythema chronicum migrans in 1913 by Lipschutz [4]. In 1930 Hellstrom associated neurological symptoms with dermatologic manifestations of the disease [5]. Nevertheless, it was not until 1976, when an outbreak of juvenile arthritis and skin rash occurred in Connecticut's city of Lyme, that LD was described [6]. Several years after, in 1982, the American entomologist Willy Burgdorfer described the causative agent of LD, a spirochete, named after him *B. burgdorferi* [7].

If not treated, LD progresses through three stages. The first stage—early localized disease—manifests by erythema migrans, which is an erythematous macule or papule that occurs one to two weeks following the tick bite and subsequently enlarges [1]. This rash can be uniformly erythematous or might have central clearing (“bull's eye”) with a median diameter around 15 cm [8]. Left untreated, *B. burgdorferi* disseminates from the site of the bite, and the disease progresses to the early disseminated stage. In this stage, which occurs three to five weeks following the initial bite, multiple (secondary) erythema migrans occur. These lesions tend to be similar to the primary erythema migrans but are usually smaller [8]. Cardiac and neurologic manifestations are also seen in the early disseminated stage, with atrioventricular heart block being the most common cardiac manifestation. Peripheral nerve palsy (particularly facial nerve) and meningitis are the most common neurological manifestations of this stage of LD. In the United States, the most common manifestation of the late disseminated stage of LD is Lyme arthritis [1, 8]. Lyme arthritis is usually mono or oligoarticular, affects large joints (knee, most commonly), and occurs weeks to months after the bite. Unlike in Europe, neurological manifestations of late LD are rare in the United States [8].

The major reservoirs for *B. burgdorferi* are birds and small mammals such as mice and chipmunks [1, 9]. While deer are not competent hosts for *B. burgdorferi*, they are essential for the *I. scapularis* life cycle. The tick *I. scapularis* has three stages of development: larva, nymph, and adult tick [1]. In North America, the life cycle of *I. scapularis* takes approximately two years to complete [9]. Egg laying usually begins in May; hence, larvae are the most abundant during the summer. These larvae feed on small mammals such as the white footed mouse during summer, at which point transmission of *B. burgdorferi* occurs. As the winter approaches, the tick larvae enter a dormant stage in which they stay throughout the winter. In the beginning of the spring of the second year, the larvae that survived the winter mold into the next stage of tick development—nymph. During the spring/summer of the second year those nymphs seek suitable hosts for feeding, including humans. Following a bloody meal, the nymphs mold into adults. If an adult tick survives the winter, it will seek another host (usually a large mammal such as deer) on which it will feed and be able to lay eggs. At that point the two year life cycle is completed [8, 9].

Nymphs are usually responsible for the majority of the infection transmission to humans. They are abundant during the spring and summer months when humans' outdoor activities are at the peak. Their small size (only few millimeters in diameter unlike common dog ticks) and the secretion of bradykininases (enzymes that break bradykinins—enzymes of inflammation) contribute to the fact that the majority of patients do not remember the tick bite [8, 10]. The risk of infection transmission from the infected tick depends on the duration of feeding. The ticks are most likely to transmit infection after a prolonged period of feeding, such as 36 hours or more. Yet, infection can be transmitted even after as little as 24 hours of feeding [10].

There is a growing body of evidence showing that climate change may affect the incidence and prevalence of certain vector-borne diseases such as malaria, dengue, West Nile fever, and LD. Unlike weather, which defines a condition of the atmosphere over a short period of time, climate represents atmospheric “behavior” over a relatively long period of time [11]. Climate change, therefore, refers to changes in long-term averages of daily weather including temperature, humidity, air pressure, and precipitation. The incidence of tick-born zoonoses such as LD is particularly likely to be affected by climate change because ticks spend the majority of their life cycle outside the host in an environment where temperature and humidity directly affect their development, activity, survival, and host-seeking behavior [12]. The number of annually reported cases of LD in the United States has sharply increased over the last three decades, from about 10,000 in 1991 to about 28,000 annually in recent years [13]. Not only did the incidence of the disease increase, but also its geographical distribution. While climate change might significantly contribute to the emergence of new infections, it is interesting to contrast this change in the incidence of tick-borne diseases in the United States with the changes happening in Europe. There, during the 9 years prior to 2015, the growth of the cases of louse-born relapsing fever (due to *B. recurrentis*) has been associated to the increase in refugees [14–16]. Furthermore, in the last few decades, newly recognized tick-borne rickettsioses have been shown to be present. *R. conorii* sub sp. *Israelensis* has been detected in human cases in Sicily and Sardinia in Italy and in different regions of Portugal [17].

This emergence of Lyme disease in the United States is at least partially attributed to climate change [12]. However, the magnitude of impact is still unclear. In this study, we investigate the effect of climatic variables on the incidence of LD in 15 U.S. states with the highest incidence of the disease. Those states contribute to 95 percent of reported cases.

## 2. Materials and Methods

We merged two types of data to conduct the fixed effects analysis in this study: annual county-level epidemiological data on LD cases from the Centers for Disease Control and Prevention (CDC) and meteorological data from the National Oceanic and Atmospheric Administration (NOAA). Both databases are publicly available.

### 2.1. Epidemiological Data

LD cases have been voluntarily reported to the CDC since 1991 by state and territorial health departments as part of the National Notifiable Disease Surveillance System (NNDSS). The annual county-level number of cases for the period 2000–2016 is publicly available at http://.cdc.gov/lyme/stats/ and is the main input for our analysis. A total of 482,297 cases were reported during that period (see the evolution of the number of cases in annual maps elaborated by the CDC, also available at http://.cdc.gov/lyme/stats/). Until 2007, a case of LD was defined as either (1) a physician-diagnosed erythema migrans rash of more than 5 cm in diameter or (2) at least one objective late manifestation (i.e., musculoskeletal, cardiovascular, or neurologic) with laboratory evidence of infection with *B. burgdorferi* (CDC 1997). The national surveillance case definition was revised in 2008 to include probable cases. State or local health departments are responsible for ensuring that cases reported to the CDC meet the case definition, and state health officials have used various methods to ascertain cases including provider-initiated passive surveillance, laboratory-based surveillance, and enhanced or active surveillance [18]. Over 95 percent of LD cases in the United States occurred in 15 states during our study period, primarily in the Northeast and Upper Midwest (Connecticut, Delaware, Maine, Maryland, Massachusetts, Minnesota, New Hampshire, New Jersey, New York, Pennsylvania, Rhode Island, Vermont, Virginia, West Virginia, and Wisconsin). These are the “high-incidence states,” where the average incidence was at least 10 confirmed cases per 100,000 persons in the previous three reporting years (see http://.cdc.gov/lyme/stats/tables.html). We focus our analysis on counties from those states and present results for the incidence of LD—cases per 100,000 population—including all cases reported during our period of analysis. Because the case definition changed in 2008, we also provide estimates based on the LD incidence reported before and after 2008. Annual population data used to calculate the LD incidence is publicly available from the U.S. Bureau of Economic Analysis (http://.bea.gov/itable/index_regional.cfm).

### 2.2. Meteorological Data

For meteorological data, we used daily measurements of maximum and minimum temperature as well as total precipitation from NOAA, publicly available at http://.ncdc.noaa.gov/cdo-web/datasets. This dataset provides detailed weather measurements at over 20,000 weather stations across the country. Daily average temperature was calculated as the arithmetic average of daily maximum and minimum temperatures, in degree Celsius (°C). Annual average temperature for the period 2000–2016 was obtained by averaging all daily observations throughout the year. For counties with no weather stations, we imputed annual average temperature by computing a weighted average of that variable from the counties within 50 miles of the original county centroid using inverse distance weights. With measures of annual county-level average temperatures in hand, indicator variables for bins of annual average temperature were generated straightforwardly. Each indicator variable takes the value one if the annual average temperature for a county is in the prespecified range, and zero otherwise. We created indicators for the following ranges: below 5, 5–7, 7–9, 9–11, 11–13, 13–15, and above 15°C. The shares of observations in each bin are reported in Table 1. Annual total precipitation for the period 2000–2016 was obtained by summing all daily precipitation for a county, in centimeters (cm). Imputation for counties with no weather stations was done as described for temperature. Indicator variables for bins of annual total precipitation were generated in a fashion similar to temperature for the following ranges: below 70, 70–120, 120–170, 170–220, 220–270, and above 270 cm. Again, the shares of observations in each bin are reported in Table 1. The average annual total precipitation for the counties in our sample is approximately 174 cm. For reference, the average annual rainfall is 20 cm in Phoenix (Arizona), 87 cm in Madison (Wisconsin), 120 cm in Providence (Rhode Island), 157 cm in Miami (Florida), and 300 cm in Mt. Rainier (Washington).

**Figure 1:**
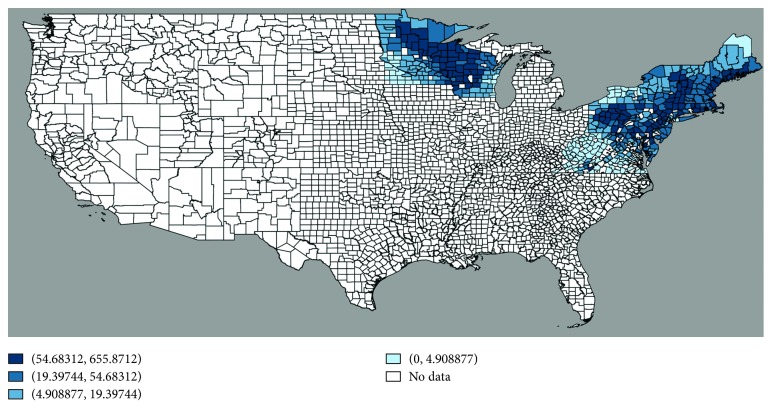
Map of the United States with highlighted counties from our Sample. Note: this map displays our sample of 468 counties in the 15 states considered by CDC as the states with the highest incidence of LD (over 95 percent of all cases in the United States). Darker blue colors represent higher incidence of the disease—cases per 100,000 population.

Once we merged the information of LD cases with climatic variables, our sample contained a balanced panel of 468 U.S. counties over the period 2000–2016.  Figure 1 displays the counties in our sample in the map of the United States, with color code based on the incidence of LD. Table 1 reports the summary statistics of our sample. Observe that most of the counties used in our sample come from Minnesota, Wisconsin, New York, Pennsylvania, Virginia, and West Virginia. Also, notice that the temperature bins with the highest shares of observations cover the range 7–13°C and the precipitation bins with the largest shares cover the range 70–220 cm.

### 2.3. Empirical Strategy

Using standard longitudinal or fixed effects methods [19–21], the typical panel regression model to examine the impact of climatic variables *C*—in our case, annual average temperature and annual total precipitation—on an outcome of interest *y*—in our case, the incidence of LD (cases per 100,000 population)—takes the form

(1) $$\begin{array}{c} y_{it}=\beta C_{it}+\gamma Z_{it}+\mu_{i}+\theta_{t}+\lambda_{\text{s}}f\left(t\right)+\varepsilon_{it}, \end{array}$$

where *i* indexes different geographic areas (in our case, counties), *t* indexes time (in our case, years), and *s* indexes a larger geographical area (in our case, states) [22]. The additional explanatory variables will be explained below, but the error process *ε* is typically modeled using robust standard errors, allowing for arbitrary correlation over time and space in the covariance matrix by clustering at the county level.

Noting that *C* varies plausibly randomly over time—i.e., “weather” draws from the county “climate” distribution—this approach resembles an experimental design and, therefore, *β* identifies the causal effect of weather shocks on the incidence of LD [22]. The fixed effects for county, *μ*_*i*_, absorb fixed spatial characteristics, whether observed or unobserved, disentangling the shock from many possible sources of omitted variable bias, such as geographic features (e.g., elevation and ruggedness) and county baseline economic characteristics (e.g., GDP, population, and number of hospital beds and number of physicians per 100,000 population) that are likely to be correlated to climatic variables. Time-fixed effects, *θ*_*t*_, further neutralize any common trends and thus help ensure that the relationships of interest are identified from idiosyncratic local shocks. State-specific time trends, *λ*_*s*_*f*(*t*), are added to allow for differential trends in subsamples of the data, controlling for a number of observed and unobserved factors affecting the outcome of interest that vary over time at the state level, such as state health expenditures and state public awareness campaigns regarding the incidence of particular diseases. In our preferred specification of Equation (1), *f*(*t*) is a quadratic function of time, that is, it includes state-specific quadratic time trends, as will be explained in more details in Results and Discussion.

It is imperative to explain the choice of temperature and precipitation as our climatic variables. One can assume that the incidence of LD might be related to tick activity. In fact, laboratory studies indicate that temperature determines whether or not, and to what extent, *I. scapularis* can move to seek hosts, whereas humidity determines how high ticks quest above ground level, where their resource for rehydration exists, and for how long they can remain actively host-seeking before retreating to rehydrate [23–25]. We use precipitation instead of the ideal measure of relative humidity because the latter is only available for half of our county-year observations. Nevertheless, in unreported analysis available upon request, we find similar results when using the subsample with information on relative humidity. Besides the biological effects of climate on tick vector abundance and activity, there may be behavioral impacts of climate on human exposure to ticks. Previous studies have found that individuals spend more time outdoors as temperature rises, up to a point where being outside becomes undesirable due to the excessive heat [26, 27]. Additionally, individuals may engage in adaptive responses to avoid exposure to ticks such as the use of deer-baiting devices to kill ticks [28].

A fundamental issue in Equation (1) is regarding the functional form of *C*. Following previous studies [29–32], we use indicator variables for bins of annual average temperature and for bins of annual total precipitation. These bins are listed in Table 1 and were described in the data section. Thus, the only functional form restriction is that the impact of the annual average temperature on the incidence of LD is constant within 2°C intervals. The choice of narrow temperature bins represents an effort to allow the data, rather than parametric assumptions, to determine the incidence-temperature relationship, while also obtaining estimates that are precise enough that they have empirical content [29–32]. This degree of flexibility and freedom from parametric assumptions is only feasible because we are using 16 years of data from a large area of the United States. Similarly, we use simple indicator variables for precipitation based on annual rainfall in county *i* in year *t*. Each indicator corresponds to a 50-cm bin, ranging from less than 70 cm to more than 270 cm.

Another important methodological decision to make when implementing panel regression models concerns the inclusion of other time-varying observables, *Z*_*it*_. Including *Z*_*it*_ may absorb residual variation, hence producing more precise estimates. However, adding more controls will not necessarily produce an estimate of *β* that is closer to the true *β*. If the *Z*'s are themselves an outcome of *C*, which may well be the case for controls such as GDP, institutional measures, and population, including them will induce an “overcontrolling problem” (in the language of the model, if *Z* is in fact *Z*(*C*), then Equation (1) would instead be written as *y* = *f*(*C*), *Z*(*C*)) and estimating an equation that included both *Z* and *C* would not capture the true net effect of *C* on *y* (again, see Dell et al. [22]). For example, suppose that poorer counties in the United States tend to be both hot and have low-quality institutions. If hot climates were to cause low-quality institutions, which in turn cause low income, then controlling for institutions in Equation (1) can have the effect of partially eliminating the explanatory power of climate, even if climate is the underlying fundamental cause. Therefore, if the incidence of LD is the outcome of interest, for example, then controlling for changes in health personnel or infrastructure would be problematic if the climatic variables influence those changes, directly or indirectly. Our preferred specification of Equation (1) does not include additional time-varying explanatory variables, but we also report separate estimates for counties above and below the U.S. median per capita income. This variable should reflect patterns of development across the nation.

## 3. Results and Discussion

Tick-borne diseases are an important public health concern and the incidence of these infections is increasing in the Unites States and worldwide [33]. Complex interactions between humans and climate change are contributing to the emergence of new diseases and the spread of already known ones to regions where they were unable to exist before. Environmental factors such as temperature and humidity have been shown to influence tick abundance, availability of hosts, their survival, and disease transmission. LD is a classic example of linkage between environmental factors and disease occurrence and spread (the U.S. Environmental Protection Agency (EPA) is actually using the number of LD cases as a climate change indicator (http://.epa.gov/climate-indicators/climate-change-indicators-lyme-disease)). For a region to be suitable for LD occurrence and transmission, the climate needs to allow the survival of both ticks and mammalian hosts necessary for completion of tick life cycle [25]. The emergence of LD in the northeast of the Unites States in 1970 was thought to be due to the expansion of the tick population associated with reforestation and expansion of the key host for tick life cycle—deer [34]. However, a recent study from Canada demonstrated the expansion of *I. scapularis* population despite deforestation [35]. Previous studies, both empirical and simulation-based, have demonstrated that a warming climate has a positive effect on the expansion of the tick population through an increase in tick survival and improved access to hosts necessary for feeding [36, 37]. Our study aimed to determine the influence of temperature and humidity on the incidence of LD within 15 U.S. states that account for the majority of reported cases.

Our estimated impacts of climatic variables on the incidence of LD—cases per 100,000 population—are reported on Table 2. Recall that the main sample contains only counties from those 15 U.S. states with the highest incidence of LD cases according to CDC. In column 1, we controlled for observed and unobserved time-varying factors affecting all sample counties equally in each year such as macroeconomic conditions and changes in health law and health expenditure at the federal level, and for observed and unobserved time-invariant factors affecting each county over the sample period such as county geographical features and historical (baseline) health infrastructure. In column 2, we added state-specific linear time trend to control for observed and unobserved changes in state variables affecting the health outcomes such as expansion of Medicaid, campaigns to raise awareness of healthy behaviors, etc. For our preferred specification in column 3, we allowed those state-specific time trends to reverse direction over time by adding quadratic terms. For example, we are controlling for observed and unobserved increases in state health expenditures in a number of years as well as decreases afterwards, or decreases in funds for campaigns raising awareness of LD, and increases in funding once more cases are confirmed. Column 3 is our preferred specification not only because the increase in the R-squared relative to previous columns indicates an improvement in the goodness-of-fit of our econometric model, but also because it takes into account important controls. In fact, the similarity in the increase in the R-squared and in the adjusted R-squared indicates that the additional explanatory variables are indeed relevant to explain the incidence of LD. Otherwise, the adjusted R-squared would have penalized our column-3 econometric specification. Both the R-squared and the adjusted R-squared reveal that our model explains over 70 percent of the variation in the incidence of LD in the United States over the period 2000–2016.

**Figure 2:**
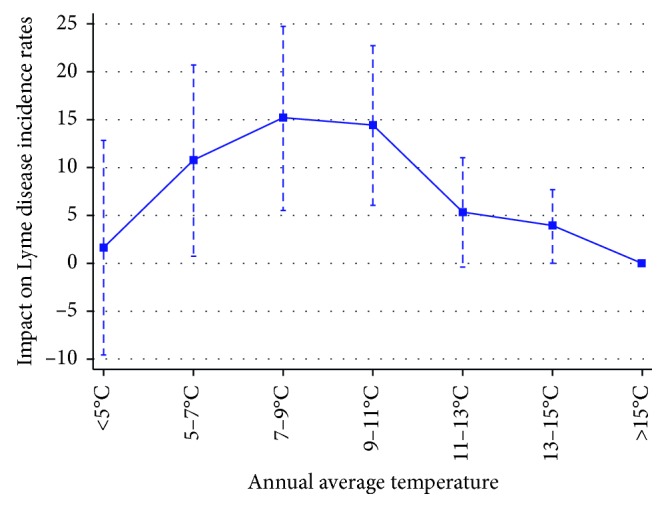
The impact of temperature on the incidence of LD. Note: this figure presents the estimated impacts of temperature on the incidence of LD—cases per 100,000 population—reported in the last column of Table 2 and represented here by blue squares. The vertical dashed lines around each blue square represent the 95 percent confidence interval.

We now describe the results from our preferred specification (Table 2, column 3). Relative to counties with annual average temperature above 15°C, counties with annual average temperature below 5°C have 1.6 additional cases of LD per 100,000 population, but this estimate is not statistically significant (not distinguishable from zero, or alternatively not distinguishable from the reference group). That estimate jumped to 10.7 cases per 100,000 population for counties with annual average temperature between 5 and 7°C, and to 15.1 for counties with annual average temperature between 7 and 9°C. Then, it stabilized for counties with annual average temperature between 9 and 11°C—14.4 cases per 100,000 population—but dropped to 5.3 and 3.9 for counties with annual average temperature between 11 and 13°C, and 13 and 15°C, respectively. We display these estimates more clearly in Figure 2, where we can see the approximately concave or inverted-U shape of the incidence of LD response to temperature.

Three features of these results are worth discussing. First, the sharp increase in the number of LD cases per 100,000 population happens in the 5–7 and 7–9°C annual average temperature bins. This might be associated with tick activity (Schulze et al. provided suggestive evidence that precipitation and temperature played a limited role in predicting the abundance of *I. scapularis* nymphs at an LD-endemic area over the period 1998–2005. Thus, we focus on tick activity in understanding our findings, as in Burtis et al.). Indeed, Duffy and Campbell [38] used flagging samples of adults *I. scapularis* through the winter to infer a minimum temperature threshold for activity of approximately 4°C. Clark [23] used laboratory experiments to determine a minimum temperature threshold for activity by adult *I. scapularis* of 9–11°C, but some individual nymphs were capable of movement and coordinated movement at much lower temperatures, 4.2 and 6.3°C, respectively. (Notice that, by definition, annual average temperature includes a range of observed temperatures throughout the year. Therefore, our temperature bins do not have to match precisely the thresholds highlighted in those studies. It is remarkable, however, that the correspondence is approximately accurate.) Second, the similarity of estimates for the ranges of annual average temperature 11–13°C, 13–15°C, and above 15°C. In fact, Vail and Smith [24] found no significant difference in the mean distance moved or time spent in questing posture for *I. scapularis* nymphs held at 10 versus 15 or 20°C. Third, the concave or inverted-U shape of the LD incidence response to temperature. Vail and Smith [24, 39] and Ogden et al. [25] found laboratory and field evidence that tick survival and activity peaked at certain temperatures, and then decreased, with peak temperatures varying considerably depending on the outcome of interest. The inverted U-shaped relationship between temperature and LD incidence is also consistent with the pattern of human exposure to ticks. In fact, temperature has been shown to have nonlinear impacts on the time adults and children allocate to outdoor activities. As temperature rises, individuals engage in more outdoor activities, but after it reaches a maximum tolerable temperature, they decrease the time spent outdoors [26, 27]. Similarly, as temperature rises, society may engage in adaptive actions to avoid exposure to ticks such as the deployment of deer-baiting devices called four-posters to kill ticks [28]. Feeder stations that resemble four-poster beds lure deers with corn. Rollers soaked with the pesticide permethrin rub the animals' necks as they eat the corn, killing ticks. As an example, this year, dozens of those devices were installed in Shelter and Fire Islands, NY, as part of a $1.2 million tick-removal effort.

While temperature is supposed to influence the extent to which *I. scapularis* can move to seek hosts, humidity is supposed to affect how high ticks quest above ground level, where resources and rehydration are available, and for how long they can remain actively host-seeking [23–25]. In our longitudinal analysis, we included bins of total precipitation during the year as a proxy for humidity. As we can see in our preferred specification (column 3) in Table 2, there was no statistical difference in the incidence of LD between counties with more than 270 cm of total precipitation—the reference group—and counties with less rainfall. This result seems to be consistent with laboratory evidence provided by Vail and Smith [39] who found no difference in the time in questing posture across any levels of relative humidity, and no difference in questing height at levels of relative humidity below 100 percent. Berger et al. [40] also found that mean weekly daytime relative humidity did not significantly predict tick activity in the field. Given such findings, we have focused on the relationship between temperature and LDs cases per 100,000 population in our discussion (it is worth mentioning that although our findings are consistent with laboratory and field evidence on tick activity and survival, and corroborate Subak's [9] findings of a weak relationship between the incidence of LD and a same-year moisture index for seven northeastern U.S. states during 1993–2001, they are different from the results found by McCabe and Bunnell; using data for seven northeastern U.S. states during the 1992–2002 period, those authors found that late spring/early summer precipitation was a significant climate factor affecting the occurrence of LD, but that temperature did not seem to explain the variability in LD reports).

Table 3 reports the impacts of climatic variables on the incidence of LD for counties above and below the U.S. median income per capita, and for cases reported before and after 2008. Although richer counties might have more resources to deal with clinical and public health issues, we did not find any statistical difference between our estimates in richer versus poorer counties. With respect to post-2008, when probable cases of LD were included along with confirmed cases, we noticed a much noisier relationship between temperature and LD incidence. This is not surprising because attenuation bias from measurement error is usually exacerbated in panel data regressions.

Our results imply that climate change will have a sizable impact on the number of cases of LD in the United States in the coming decades. Using the estimated impacts of temperature on the incidence of the disease in Table 2, column 3, and the distribution of county-year observations in each bin of annual average temperature from Table 1, we have predicted an increase of 8.6 cases of LD per 100,000 population per county-year, an increase of about 21 percent relative to the average incidence of the disease over the period 2000–2016. This was done by assuming a 2°C increase in annual average temperature in the northern area of the United States by mid-century (2036–2065). (The 2°C increase in annual average temperature implies that the share of counties in one 2°C bin in Table 1 would show up in the following 2°C bin. For example, the 26 percent of counties in the 7–9°C temperature bin in Table 1 would show up in the 9–11°C bin. The calculation of the impact of that increase in annual average temperature then used the estimates reported in Table 2, column 3, and the shift up of the shares presented in Table 1.) This temperature increase is assumed to be an approximation for the change the region we focus on might experience in the future. In fact, it is slightly below the mid-century (2036–2065) projections for the Northeast (2.21°C or 3.98°F) and Midwest (2.34°C or 4.21°F) from the Fourth National Climate Assessment (NCA4) (USGCRP 2017). This is under the more conservative Representative Concentration Pathway (RCP) 4.5, which assumes global annual greenhouse gas emissions peaking around 2040, then declining. Under the RCP 8.5, which assumes that emissions will continue to rise throughout the twenty-first century, those mid-century (2036–2065) predicted increases in annual average temperature would be 2.83°C (5.09°F) for the Northeast and 2.94°C (5.29°F) for the Midwest (see definition of the NCA4 regions at scenarios.globalchange.gov/regions_nca4).

Given the increase of 8.6 cases of LD per 100,000 population per county-year associated with a 2°C increase in temperature and the average population for a county-year in our sample of 149,606 persons, we have predicted an increase in the number of LD cases by approximately 6,040 per year in the counties in our sample (again, they represent over 95 percent of the cases in the entire country). Because the average annual number of LD cases in United States over the period 2000–2016 was 28,370, that amount translates into an increase of roughly 21 percent in the number of LD cases in the coming decades.

## 4. Conclusion

In this study, we have shown that a sizable increase in the incidence of LD cases in endemic areas of the United States due to climate change is imminent. These findings should alert public health agencies, physicians, and patients. On the one hand, better education and increased awareness among patients and physicians is important because early recognition and treatment are usually highly effective in preventing debilitating consequences of untreated Lyme disease and the potential post-Lyme syndrome. On the other hand, public health authorities should be alert to work on strategies to limit tick and host population and consequently decrease the incidence of LD not only in endemic areas, but also in neighboring locations where the disease has only been sporadically reported, or not reported at all. In fact, climate change may make those areas suitable for the establishment of tick and host populations.

Our study has a number of limitations. First, because of data limitations, we have used annual data to examine the relationship between the incidence of LD and climatic variables. Thus, we were unable to address the seasonality of LD cases throughout the year, as highlighted by Moore et al. [41] and the climate change influences on the annual onset of LD, as studied by Monaghan et al. [42]. Second, we have focused our analysis on counties from the highest incidence states, regardless of the spatial distribution of blacklegged ticks. Hence, we cannot comment on whether these reported cases were autochthone—most likely the vast majority of the cases—or imported. In an ongoing research project, we are examining the climate-LD incidence relationship over U.S. counties with established tick population versus counties with ticks reported, but not yet established. Third, although we have overcome a number of omitted variable bias issues with the fixed effects analysis, we have not scaled our results relative to areas with few LD cases. In work still in progress, we are using a border approach to compare our estimates for the places with high incidence of LD with estimates for their corresponding neighboring areas.

## Figures and Tables

**Table 1:** Summary statistics from our sample.

Variable	Obs.	Mean	Std. dev.	Min.	Max.
Incidence of Lyme disease	7,956	41.75	76.24	0	1581.15
Avg. temp.: below 5°C	7,956	0.07	0.25	0	1
Avg. temp.: 5–7°C	7,956	0.16	0.36	0	1
Avg. temp.: 7–9°C	7,956	0.26	0.44	0	1
Avg. temp.: 9–11°C	7,956	0.19	0.39	0	1
Avg. temp.: 11–13°C	7,956	0.18	0.38	0	1
Avg. temp.: 13–15°C	7,956	0.11	0.31	0	1
Avg. temp.: above 15°C	7,956	0.03	0.18	0	1
Total prcp.: below 70 cm	7,956	0.05	0.22	0	1
Total prcp.: 70–120 cm	7,956	0.20	0.40	0	1
Total prcp.: 120–170 cm	7,956	0.27	0.44	0	1
Total prcp.: 170–220 cm	7,956	0.22	0.42	0	1
Total prcp.: 220–270 cm	7,956	0.15	0.35	0	1
Total prcp.: above 270 cm	7,956	0.10	0.31	0	1
Connecticut	7,956	0.01	0.12	0	1
Delaware	7,956	0.01	0.08	0	1
Maine	7,956	0.03	0.18	0	1
Maryland	7,956	0.04	0.19	0	1
Massachusetts	7,956	0.03	0.17	0	1
Minnesota	7,956	0.17	0.37	0	1
New Hampshire	7,956	0.02	0.14	0	1
New Jersey	7,956	0.04	0.20	0	1
New York	7,956	0.12	0.32	0	1
Pennsylvania	7,956	0.12	0.33	0	1
Rhode Island	7,956	0.01	0.09	0	1
Vermont	7,956	0.03	0.17	0	1
Virginia	7,956	0.12	0.33	0	1
West Virginia	7,956	0.10	0.29	0	1
Wisconsin	7,956	0.15	0.35	0	1

Note: this table presents the summary statistics regarding the sample used in our, not or analysis. Our sample contains 7,956 observations from 468 counties over 17 years (2000–2016). Those counties are from the 15 states considered by the CDC as the states with the highest incidence of LD (over 95 percent of all cases in the United States). All variables with the exception of the incidence of LD are indicator variables taking the value of one if the statement on the far left is valid, and zero otherwise. Hence, the means for those indicator variables represent shares of the total number of observations. For example, 26 percent of the county-year observations have annual average temperature between 7 and 9°C, and 17 percent of the county-year observations come from Minnesota.

**Table 2:** The impacts of temperature and precipitation on the incidence of LD.

Main results			
Dep. var.: LD incidence	(1)	(2)	(3)
Avg. temp.: below 5°C	7.9101 (6.1820)	4.9673 (5.9583)	1.6156 (5.7073)
Avg. temp.: 5–7°C	17.2713^*∗∗∗*^ (5.6208)	13.3615^*∗∗*^ (5.4461)	10.7294^*∗∗*^ (5.0919)
Avg. temp.: 7–9°C	21.3359^*∗∗∗*^ (5.2593)	16.2189^*∗∗∗*^ (5.2152)	15.1306^*∗∗∗*^ (4.8862)
Avg. temp.: 9–11°C	19.9290^*∗∗∗*^ (4.3244)	15.4690^*∗∗∗*^ (4.5382)	14.4033^*∗∗∗*^ (4.2444)
Avg. temp.: 11–13°C	9.7629^*∗∗∗*^ (2.9059)	6.5636^*∗∗*^ (3.1989)	5.3232^*∗*^ (2.9025)
Avg. temp.: 13–15°C	6.6229^*∗∗∗*^ (2.0306)	4.7761^*∗∗*^ (2.1972)	3.8847^*∗∗*^ (1.9730)
Reference: above 15°C	*0*	*0*	*0*
Total prcp.: below 70 cm	13.0452^*∗∗*^ (5.4358)	13.5556^*∗∗∗*^ (4.5898)	4.6664 (4.5738)
Total prcp.: 70–120 cm	10.7113^*∗∗*^ (4.7829)	11.8325^*∗∗∗*^ (4.0619)	4.2597 (3.9230)
Total prcp.: 120–170 cm	8.1580^*∗∗*^ (4.1505)	10.4118^*∗∗∗*^ (3.6474)	5.3288 (3.4688)
Total prcp.: 170–220 cm	3.7551 (3.1761)	6.3591^*∗∗*^ (2.8603)	3.6003 (2.6573)
Total prcp.: 220–270 cm	1.8596 (2.8608)	2.3633 (2.8913)	1.2022 (2.8126)
Reference: above 270 cm	*0*	*0*	*0*

Year fixed effects	Yes	Yes	Yes
County fixed effects	Yes	Yes	Yes
Linear trend by state		Yes	Yes
Quadratic trend by state			Yes

Observations	7,956	7,956	7,956
*R* ^2^	0.6771	0.7068	0.7226
Adjusted *R*^2^	0.656	0.687	0.703

Note: this table presents the estimated impacts of climatic variables on the incidence of LD–cases per 100,000 population. Avg. temp. is annual average temperature, and total prcp. is annual total precipitation. Robust standard errors clustered at the county level are reported in parentheses. ^*∗∗∗*^Significance at 1 percent; ^*∗∗*^significance at 5 percent; ^*∗*^significance at 10 percent.

**Table 3:** The impact of climatic variables by income per capita and before vs. after 2008.

Heterogeneity				
Dep. var.: LD incidence	<PCI median	≥PCI median	<2008	≥2008
	(1)	(2)	(3)	(4)

Avg. temp.: below 5°C	9.8421 (7.2752)	6.5755 (8.5056)	−0.0423 (4.9830)	−6.0330 (6.8374)
Avg. temp.: 5–7°C	16.1789^*∗∗∗*^ (6.1200)	12.2321 (7.7779)	5.9658 (3.9566)	3.3523 (5.7814)
Avg. temp.: 7–9°C	20.0468^*∗∗∗*^ (7.2075)	12.3422 (7.5295)	6.7271^*∗*^ (3.9568)	2.3292 (4.9421)
Avg. temp.: 9–11°C	17.6905^*∗∗∗*^ (6.2141)	15.5235^*∗∗*^ (7.2675)	5.3618^*∗∗*^ (2.5961)	6.0453 (4.2411)
Avg. temp.: 11–13°C	7.5071^*∗∗*^ (3.0650)	8.2686 (5.6081)	2.7109 (1.8782)	−0.6243 (2.5552)
Avg. temp.: 13–15°C	6.1809^*∗∗*^ (2.3800)	5.3997 (3.3455)	2.7591^*∗∗*^ (1.2852)	−0.8361 (2.2234)
Reference: above 15°C	*0*	*0*	*0*	*0*
Total prcp.: below 70 cm	1.8066 (7.2816)	11.1090 (7.5837)	−5.7405 (5.5240)	−2.8211 (7.2106)
Total prcp.: 70–120 cm	0.0188 (6.8925)	9.6526 (6.5876)	−6.3607 (5.3531)	−3.5179 (6.2751)
Total prcp.: 120–170 cm	1.7949 (6.0951)	8.7628 (5.8681)	−2.0996 (4.7471)	−5.4875 (5.2265)
Total prcp.: 170–220 cm	−0.4551 (4.4063)	8.0211^*∗*^ (4.4954)	−3.3351 (4.2346)	−5.8981 (3.8901)
Total prcp.: 220–270 cm	−2.3741 (3.0281)	6.2257 (3.8928)	−1.5489 (2.9620)	−4.7632 (3.4002)
Reference: above 270 cm	*0*	*0*	*0*	*0*

Year fixed effects	Yes	Yes	Yes	Yes
County fixed effects	Yes	Yes	Yes	Yes
Linear trend by state	Yes	Yes	Yes	Yes
Quadratic trend by state	Yes	Yes	Yes	Yes

Observations	3,978	3,978	3,744	4,212
*R* ^2^	0.7804	0.7658	0.8565	0.8237
Adjusted *R*^2^	0.750	0.738	0.830	0.795

Note: this table presents the estimated impacts of climatic variables on the incidence of LD—cases per 100,000 population—by counties above and below the U.S. per capita income (PCI), and by cases before and after 2008, when probable cases of LD were included in the total number of cases in each county. Avg. temp. is annual average temperature, and total prcp. is annual total precipitation. Robust standard errors clustered at the county level are reported in parentheses. ^*∗∗∗*^Significance at 1 percent; ^*∗∗*^significance at 5 percent; ^*∗*^significance at 10 percent.

## Data Availability

The data used to support the findings of this study are included within the article.
